# Editorial: Innovative insights into pattern recognition and signaling in innate immunity

**DOI:** 10.3389/fimmu.2026.1831675

**Published:** 2026-04-01

**Authors:** Francesca Granucci, Uday Kishore

**Affiliations:** 1 Department of Biotechnology and Biosciences, University of Milano Bicocca, Milan, Italy; 2 Department of Veterinary Medicine (CAVM) and Zayed Centre for Health Sciences, United Arab Emirate University, Al-Ain, United Arab Emirates

**Keywords:** disease mechanism, innate immunity, pattern recognition, signalling, therapeutic modulation, tissue microenvironment

Innate immunity is the first and most immediate layer of host defense; however, it is not a simple or fixed system. It is a dynamic network in which target recognition, signaling, cell-cell communication, tissue context, and metabolic adaptation are tightly linked. Over the last years, major progress has been made in defining how innate immune cells and tissue-resident cells detect pathogens and danger signals, how these signals are integrated, and how they shape inflammatory or protective responses. At the same time, it has become increasingly clear that many innate immune pathways operate beyond their classical definitions. Sensors once considered restricted to one compartment can function in others; soluble molecules can behave as pattern-recognition effectors, while signaling pathways classically linked to infection also contribute to tissue homeostasis, chronic inflammation, and immune-mediated disease. The articles collected in this Research Topic reflect this broader and evolving view of innate immunity. Together, they show that progress in the field now depends not only on identifying new receptors or pathways, but also on understanding where these pathways act, how they interact with one another, and how they may be translated into clinical benefit.

A central theme emerging from this Research Topic is the continuing expansion of the pattern-recognition landscape. Several contributions revisit well-known innate sensors but place them in unexpected biological settings. In the study on HSV-2 infection (Goel et al), nuclear cGAS is proposed to cooperate with IFI16 in sensing viral DNA, promoting IFN-β production while also intersecting with inflammasome activation, autophagy, and DNA damage responses. This work extends the discussion on DNA sensing beyond the cytosol and supports a more integrated view of antiviral signaling during early infection. In another study by Majithia et al. RIG-I, classically associated with antiviral RNA sensing, was shown to drive protective type I interferon responses in glial cells challenged with bacterial pathogens. These findings are important because they place an RNA sensor in the context of bacterial meningitis and indicate that RIG-I-dependent responses in resident CNS cells may directly restrict bacterial burden. Likewise, Chakraborty et al. offer a new hypothesis for how inflammatory signaling may be initiated during Mpox infection. Together, these studies illustrate a field that is moving away from rigid receptor-pathogen categories and toward a more flexible model of innate recognition.

A second major theme is that signaling should be understood not only as a linear cascade downstream of receptor engagement, but as a layered process shaped by localization, amplification, and crosstalk. This is particularly evident in a study by Sui et al. By identifying cytoplasmic ATM as a mediator of Mn-induced TBK1 activation and antiviral cytokine production, this work broadens the functional space of a kinase traditionally linked to DNA damage repair. It also reinforces the concept that innate immune signaling can be regulated by metabolic and micronutrient-dependent mechanisms. Related ideas emerge from the study of glial RIG-I signaling and from the HSV-2 work, both of which highlight that IFN pathways are closely connected to inflammasome activity and other stress-response pathways. These articles collectively argue that the outcome of innate sensing depends not only on which receptor is engaged, but also on how signaling modules are assembled in time and space.

This Research Topic also emphasizes that innate immunity is not restricted to membrane-bound or cytosolic receptors in classical immune cells. Soluble and tissue-associated mediators remain central to immune surveillance. Shamim et al. revisit structure-function relationship of an important collectin, surfactant protein D (collagen-containing C-type lectin) as much more than a pulmonary surfactant component, presenting it as a multifunctional surveillance molecule that recognizes pathogens and allergens, shapes innate and adaptive immunity, and may also have therapeutic relevance in infection, allergy, pregnancy and cancer. In parallel, Pegoraro et al. highlight how expression of a key innate effector i.e. C1q, the first subcomponent of the classical pathway of complement activation, is controlled by coordinated epigenetic mechanisms, with important implications for inflammation, tumor biology, and tissue-specific immune regulation. C1q has a range of functions outside classical pathway activation as a charge pattern recognition innate immune molecule. C1q is also implicated in a number of disease mechanisms, and thus, understanding ways to modulate epigenetic regulation of C1q expression by innate immune cells such as macrophages and dendritic cells will have a broad ranging implications in translational medicine. Complement-related innate recognition is also explored by Kishore et al., where factor H and properdin are described as soluble pattern-recognition molecules able to interact directly with viral components and differentially influence viral entry and inflammatory responses. Properdin is the only known up-regulator of the complement alternative pathway, while factor H is a negative regulator of the alternative pathway. As mentioned with respect to C1q, properdin and factor H can also act as pattern recognition humoral factors beyond their roles as regulators of the complement system. These papers remind us that innate immunity is also organized through extracellular recognition systems and locally produced mediators that influence the earliest stages of host-pathogen interaction.

Another strength of the Research Topic is its attention to cellular communication and immune regulation. Wu et al. highlight that innate immune responses are coordinated not only through cytokines and chemokines, but also through direct intercellular exchange of signals, metabolites, and even organelles. This perspective is particularly valuable because it shifts attention from isolated cells to multicellular response networks. A related regulatory dimension is reported by Yu et al. Rather than viewing neutrophils only as terminal effector cells, this work places them within homeostatic immune circuits. Similarly, Yoshikawa et al. identify an inhibitory role for a C-type lectin receptor, Dcir, in antifungal defense by limiting neutrophil degranulation. These contributions highlight that successful innate immunity requires not only activation, but also calibration and restraint.

The clinical and translational relevance of innate immune signaling is another recurring message. This is clear in a review by Chen et al., which discusses how cholangiocytes, bile components, and tissue innate immune cells together create a local immune environment that may sustain chronic inflammation and fibrosis. It is also evident in a study by Singh et al., where IVIG treatment is associated with remodeling of innate immune cell communication and induction of distinct autophagy programs across cell subsets. More broadly, Wang et al. have identified innate immune-related biomarkers and signaling pathways linked to disease progression. Although these studies examine different diseases, they converge on the point that innate pathways are not only triggers of acute antimicrobial defense, but also determinants of chronic inflammatory states and treatment response.

The Research Topic also extends the concept of innate immunity across biological scales. A Mini-Review by Wang et al. describes how viruses evade bacterial defense systems by sequestering immune second messengers, revealing striking parallels with immune countermeasures seen in higher organisms. At the other end of the spectrum, Retnakumar et al. show that IL-33 and IL-3 can induce CD25 expression without productive IL-2 signaling, suggesting that receptor expression alone may not predict functional competence and pointing to a potential biomarker in severe COVID-19. Such studies broaden the conceptual boundaries of the field and encourage comparison across systems, species, and disease contexts.

Taken together, the articles in this Research Topic show that innovation in innate immunity now lies in integration. Pattern recognition can no longer be considered separately from signaling context, tissue microenvironment, metabolic state, or therapeutic modulation. Several contributions identify new candidate mechanisms, while others synthesize emerging concepts and reveal common principles across infection, inflammation, and immune-mediated diseases. The challenge ahead will be to connect molecular details with physiological relevance and, ultimately, with clinical application. This will require combining mechanistic studies with systems-level approaches, careful attention to cell and tissue specificity, and stronger bridges between experimental immunology and translational medicine. The work gathered here provides valuable examples of this direction. Innate immunity remains a foundational area of research, but it is also a rapidly changing one. The studies assembled in this Topic make it clear that its future will be defined not simply by discovering more components, but by understanding how recognition and signaling are organized into functional, adaptable, and clinically meaningful immune networks in a temporal and spatial manner, especially in the context of tumour microenvironment and placenta.

By bringing together studies on antiviral and antibacterial sensing, soluble immune mediators, intercellular communication, immune regulation, and translational relevance, this Research Topic offers an updated view of innate immunity as a flexible and interconnected system. These contributions not only deepen our mechanistic understanding but also point to new opportunities for therapeutic intervention. We hope that this Research Topic will stimulate further work aimed at linking molecular discovery with physiological relevance and clinical application.

